# The Impact of Mean Arterial Pressure and Volume Contraction in With Acute Ischemic Stroke

**DOI:** 10.3389/fneur.2022.766305

**Published:** 2022-03-08

**Authors:** Mona N. Bahouth, Deanna Saylor, Argye E. Hillis, Rebecca F. Gottesman

**Affiliations:** ^1^School of Medicine, Johns Hopkins University, Baltimore, MD, United States; ^2^School of Nursing, Johns Hopkins University, Baltimore, MD, United States; ^3^School of Public Health, Johns Hopkins University, Baltimore, MD, United States

**Keywords:** hydration, blood pressure, stroke severity, acute ischemic stroke, hospital care, early recovery

## Abstract

**Background and Purpose:**

Hydration at the time of stroke may impact functional outcomes. We sought to investigate the relationship between blood pressure, hydration status, and stroke severity in patients with acute ischemic stroke (AIS).

**Methods:**

We evaluated hydration status, determined by blood urea nitrogen (BUN)/creatinine ratio, in consecutive patients with AIS from a single comprehensive stroke center. Baseline mean arterial pressure (MAP) was analyzed using a linear spline with a knot at 90 mmHg. Baseline stroke severity was defined based on admission NIH Stroke Scale scores (NIHSSS) and MRI diffusion-weighted imaging.

**Results:**

Among 108 eligible subjects, 55 (51%) presented in a volume contracted state. In adjusted models, in the total sample, for every 10 mmHg higher MAP up to 90 mmHg, NIHSSS was 2.8 points lower (*p* = 0.053), without further statistically significant association between MAP above 90 and NIHSSS. This relationship was entirely driven by the individuals in a volume contracted state: MAP was not associated with NIHSSS in individuals who were euvolemic. For individuals in a volume contracted state, each 10 mmHg higher MAP, up to 90 mmHg, was associated with 6.9 points lower NIHSSS (95% CI −11.1, −2.6). MAP values above 90 mmHg were not related to NIHSSS in either dehydrated or euvolemic patients.

**Conclusions:**

Lower MAP contributes to more severe stroke in patients who are volume contracted, but not those who are euvolemic, suggesting that hydration status and blood pressure may jointly contribute to the outcome. Hydration status should be considered when setting blood pressure goals for patients with AIS.

## Introduction

Despite advances in stroke care and identification of acute therapies for ischemic stroke, stroke remains a leading cause of adult disability. (1). Blood pressure management is a critical component of patient management in the early stroke recovery period. A growing body of evidence suggests that patients who are dehydrated, volume contracted, or both at the time of stroke have worse functional outcomes independent of age, size of the stroke, or presence of complex comorbidities (2, 3). The mechanism behind this relationship is yet unknown, though many hypothesize that the worse outcome is due to blood pressure variations that alter cerebral perfusion during a period of disrupted autoregulation. We propose that hydration status may play an independent role.

Surprisingly, rehydration after stroke has received little attention in the United States outside of a small study of induced hypertension and a series of hemodilution studies (4–7). Expert consensus drives the current acute stroke recommendation for rehydration for patients who are in a volume contracted state (8). These are challenging to implement as there is no single objective measure for dehydration and the duration of therapy is not specified especially in the setting of comorbid conditions (9). There are several approaches to quantifying hydration status with varying levels of data to support such practices: non-invasive cardiac output monitoring with passive leg raise and fluid challenge, bioelectric impedance vector analysis, and serum markers of hydration status (10–14). In this study, we use blood urea nitrogen (BUN) to creatinine ratio as a surrogate objective marker of hydration status with the threshold for volume contracted state as BUN/creatinine > 15 since it is readily available for use globally (15–17). We sought to explore the relationship between hydration status, blood pressure, and stroke severity in order to test the relative contributions of these physiologic factors in patients with acute ischemic stroke (AIS). Specifically, the present study sought to determine whether or not hydration status modifies the relationship between blood pressure and stroke severity. We additionally wished to identify similar relationships between hydration and blood pressure and longer-term functional outcome, measured at 3 months using modified Rankin Scores (mRS).

## Methods

### Data Source

Consecutive patients with ischemic stroke were prospectively screened for eligibility between 2014 and 2015. Subjects were included if they had MRI-confirmed ischemic stroke within 12-h from stroke onset and did not have signs of active infection, gastrointestinal bleeding, or chronic kidney disease. Indirect measures of volume status including BUN/creatinine ratio were collected with a threshold of BUN/creatinine ratio > 15 sets as the indicator of a volume contracted state. Stroke severity was determined by the NIH Stroke Scale (NIHSSS) at the time of hospital presentation. Mean arterial pressure (MAP) was calculated using standard equations based on the first blood pressure obtained on arrival to the hospital. Infarct volume was calculated using baseline MRI diffusion-weighted imaging (DWI) sequences and diffusion to perfusion (PWI) mismatch calculated if perfusion study was obtained as the standard of care upon admission to the hospital using OleaSphere software (OleaSphere 3; Olea Medical, La Ciotat, France) by a single primary rater blinded to both diagnosis and hydration status. The study was approved by the university's Institutional Review Board.

### Statistical Analysis

Statistical analysis was performed using Stata Statistical software version 13 (StataCorp LP, College Station, TX, USA). The primary outcome was stroke severity defined by NIHSSS. Comparisons of key clinical characteristics based on hydration status and MAP were made using Fisher's exact tests for categorical variables and the *t*-tests for continuous variables. Given the appearance during descriptive analysis using a LOWESS curve of a non-linear pattern between MAP and NIHSSS, a linear spline was used, with a single knot at 90 mmHg.

Statistically significant variables derived from univariable analysis as well as other variables that may be plausibly associated with stroke severity were considered as covariates; however, final model selection was based on the Akaike Information Criterion. Multivariable linear regression models evaluating MAP using this spline, and considering NIHSSS as the dependent variable, were adjusted for age, sex, atrial fibrillation, diabetes, and infarct volume. Exploratory analysis of the functional outcome was based on an a priori dichotomized mRS: 0–1 = favorable functional outcome and > 1 = poor outcome, in similarly-adjusted logistic regression models.

To investigate the relationship between hydration status and blood pressure at the time of stroke, we used the above-described linear regression models, adjusted for the same potential confounders, evaluating the effect of MAP on baseline NIHSSS, but stratified models by hydration status, along with formal testing for interaction between MAP and hydration status. Univariate analysis and multivariable logistic regression models were used to evaluate dichotomized 3-month outcomes (mRS 0–1 or mRS > 1) by hydration status and MAP.

## Results

A total of 312 subjects were screened for this study. Among those 185 subjects were excluded for reasons of presentation to the hospital > 12 h from stroke onset, unable to complete MRI or presence of baseline renal disease. Of the 126 subjects remaining, 108 had sufficient labs and baseline blood pressure data for analysis of which 55 (51%) were in a volume contracted state at the time of hospital presentation. Mean initial MAPs in the volume contracted and euvolemic groups were 112.3 mmHg and 112.5 mmHg respectively (*p* = 0.97). With this sample size, we have 80% power to detect a difference between groups with alpha 0.05 (Table 1).

**Table 1:** Demographics for the analytic sample.

		**Total sample stratified by BUN/ creatinine status (*N =* 108)**			**Total sample stratified by initial MAP** **(***N =* **108)**		
	**All (*N =* 108)**	**Normal BUN/creatinine (*N =* 53)**	**Elevated BUN/creatinine (*N =* 55)**	***P* value**	**MAP <90 mmHg (*N =* 18)**	**MAP > 90 mmHg (*N =* 90)**	***P* value**
Age (years) (Mean ± SD)	60 ± 14	58	62	0.12	57	60	0.34
Sex (Female) *N* (%)	48 (44%)	19 (36%)	29 (53%)	0.09	8 (44%)	40 (44%)	1.00
Atrial fibrillation *N* (%)	15 (14%)	10 (19%)	5 (9%)	0.17	3 (17%)	12 (13%)	0.71
Hypertension *N* (%)	83(77%)	44 (83%)	39 (71%)	0.17	11 (61%)	72 (80%)	0.12
Diabetes *N* (%)	35 (32%)	15 (28%)	20 (36%)	0.42	5 (28%)	30 (33%)	0.79
Ejection Fraction below 50%^*^N (%)	19 (18%)	11 (22%)	8 (15%)	0.44	4 (24%)	15 (17%)	0.51
Acute revascularization therapy^†^ *N* (%)	25 (23%)	14 (26%)	11 (20%)	0.43	3 (17%)	22 (24%)	0.56
Serum Sodium (mean ± SD)	140 ± 3	140.5	139.6	0.15	139	140	0.12
Baseline Hemoglobin	13.4 (2.2)	13.7	13.1	0.11	13.4	13.4	0.07
Length of stay (days) (mean ± SD)	6.1 ± 6.2	5.6	6.5	0.47	6.0	6.1	0.95
Initial NIHSS (mean ± SD)	6.0 ± 5.4	6.4	5.7	0.48	7.7	5.7	0.27
Lesion volume (cc)^‡^	9.8 ±19.6	10.8	8.9	0.61	14.9	8.8	0.43
DWI:PWI mismatch present^**^	62 (73%)	29 (71%)	33 (75%)	0.81	7 (64%)	55 (74%)	0.48

* *Ejection Fraction available on N = 103*.

† *(Intravenous tPA and/or endovascular therapy)*.

‡ *Lesion volume available on N = 108;*

** *DWI:PWI mismatch defined as > 1.2; N = 85*.

**Figure 1:**
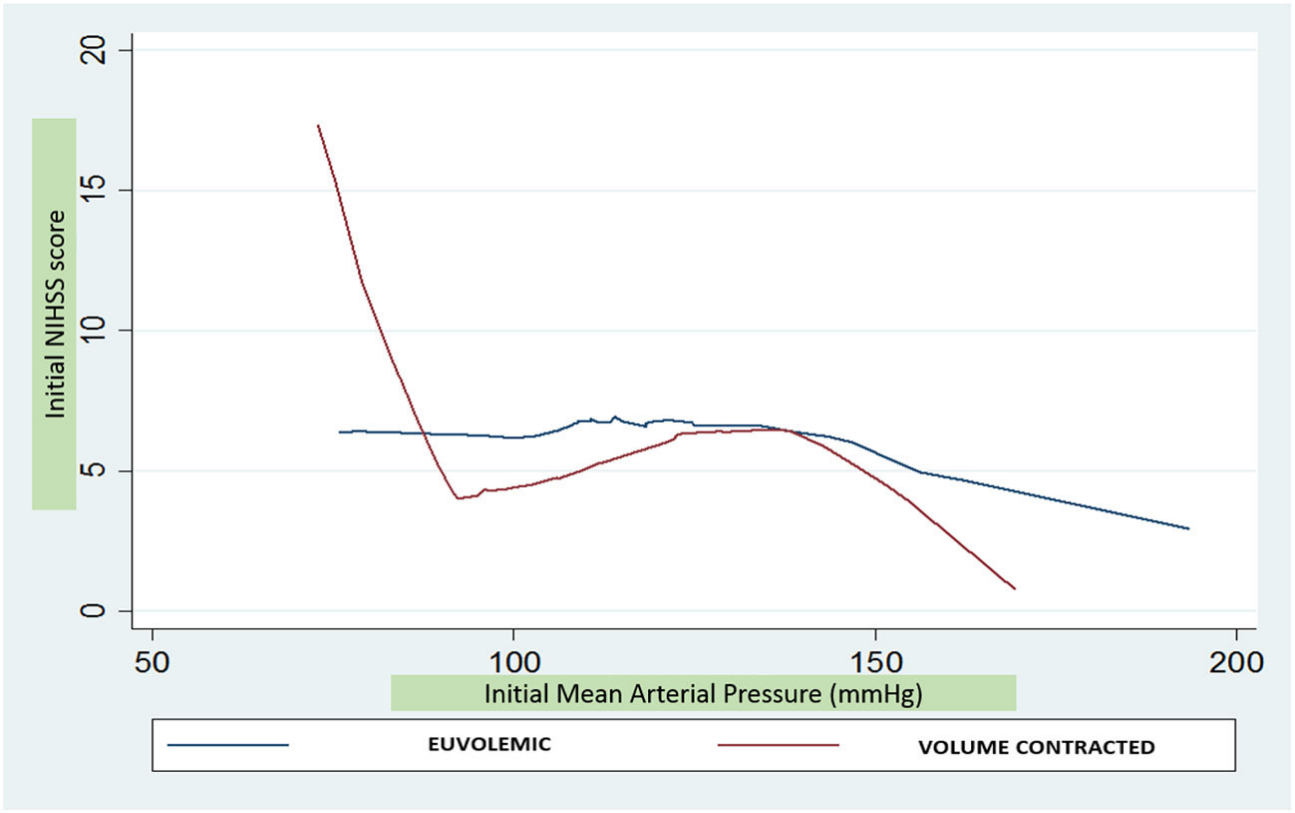
Comparison of NIH stroke scale score and mean arterial pressure by hydration status.

In adjusted models including the total sample, for every 10 mmHg higher MAP up to 90 mmHg, NIHSSS was 2.8 points lower (*p* = 0.053), without further statistically significant association between MAP above 90 mmHg and NIHSSS (Table 2). When analyses were stratified, however, it was apparent that this relationship was entirely driven by the individuals in a volume contracted state: MAP was not associated with NIHSSS in individuals who were euvolemic. For individuals in a volume contracted state, each 10 mmHg higher MAP, up to 90 mmHg, was associated with 6.9 points lo8wer NIHSSS (95% CI −11.1, −2.6; *p* = 0.002) (Figure 1). The formal test for interaction between MAP and dehydration status for MAP values below 90 mmHg was statistically significant (*p* = 0.01). For subjects with initial MAP above 90 mmHg, there was no association between increased MAP and severity in the volume contracted or euvolemic groups. There was no difference in odds of poor 3-month outcome mRS based on differences in initial MAP (unadjusted OR 1.05; 95% CI 0.85, 1.29).

**Table 2:** Effect of mean arterial pressure (per 10 mm Hg) on stroke severity and outcome: multivariable regression analysis.

	**Stroke severity: baseline NIHSS score** (***N** **=*** **108)**			**Functional outcome: 3 month modified rankin scale (mRS** **>** 1 vs. mRS 0–1)^**^		
	***N***	**Adjusted beta^§^(95% CI) for MAP up to 90 mm Hg^#^**	**Adjusted beta^§^(95% CI) for MAP above 90 mm Hg**	***N***	**Adjusted OR^§^(95% CI) for MAP up to 90 mmHg**	**Adjusted OR^§^(95% CI) for MAP above 90 mmHg**
All patients	108	−2.8 (−5.6, 0.04)	−0.1 (−0.3, 0.5)	86	0.25 (0.02, 3.42)	1.11 (0.86, 1.44)
Volume contracted patients	55	−6.9 (−11.1, −2.6)	0.3 (−0.4, 1.1)	45	0.38 (0.004, 40.76)	1.45 (0.86, 2.43)
Euvolemic patients	53	1.3 (−3.0, 5.5)	−0.1 (−0.7, 0.4)	41	0.25 (0.007, 8.93)	0.98 (0.69, 1.39)

§ * Adjusted for age, gender, atrial fibrillation, diabetes, and lesion volume*.

# *p-interaction for MAP within this range and dehydration status = 0.01*.

** *3 month mRS dichotomized a priori as bad outcome (mRS > 1) vs. good outcome (mRS 0–1)*.

In terms of MRI findings, the size of the stroke lesion was measurable in all 108 subjects. The average ischemic lesion size based on diffusion-weighted imaging was 9.8 ± 19.6 cc. In this cohort, 84/108 (78%) subjects had perfusion imaging sufficient for interpretation if diffusion to perfusion mismatch. There was no difference in diffusion to perfusion mismatch based on hydration status nor mean arterial pressure.

## Discussion

Although limited by a relatively small sample size, these data suggest that lower MAP may be an important variable in subjects who are volume contracted at the time of stroke. It suggests that volume contracted patients with ischemic stroke may be at higher risk for more severe stroke, particularly if they also have low blood pressure. Notably, there was no significant difference between blood pressures in the volume contracted and euvolemic patients.

A large percentage of patients with AIS are volume contracted at the time of stroke (2–4, 15, 16). Intravascular volume is one component in blood pressure and avoidance of low blood pressure has been standard in acute stroke care (8). These data suggest that avoidance of low blood pressure may be especially important in patients who are dehydrated. Therefore, hydration status may be an important consideration when setting early blood pressure goals.

This study also expands on the knowledge generated from earlier studies of induced hypertension as a potentially helpful adjunct therapy after ischemic stroke (18–20). These studies used a combination of pharmacological agents and volume expanders to elevate blood pressure at the time of stroke and demonstrated improved clinical outcomes. Our results suggest an opportunity for potential benefit using a simpler treatment using solely intravenous saline. This is especially important given that the largest burden of stroke worldwide is in low-resourced settings, and intravenous saline could represent a scalable intervention to improve stroke outcomes even in these settings.

There are multiple potential reasons for hydration and stroke severity to be linked, independent of MAP. First, blood viscosity may be impacted by hydration, such that lower viscosity improves perfusion. Next, patients who are chronically volume contracted may have more accumulation of brain disease prior to the stroke event thus impacting severity. Finally, comorbidities such as diabetes and heart failure might be associated with volume contraction and influence outcome. This study further clarifies the relationship between hydration status, blood pressure, and stroke severity. That is, the combination of low blood pressure and volume contraction is more strongly associated with higher stroke severity than either alone.

This study has several limitations, including those common to the small sample size. Further, only 80% of these subjects had 3 month mRS completed limiting our ability to assess functional outcomes in the entire cohort. Next, data collected within the standard care environment are subject to variation in technique, specifically when related to the measurement of initial blood pressure. Nevertheless, these data yield the important finding that blood pressure and hydration status are potentially independent factors contributing to stroke severity, independent of infarct volume, and suggest that dehydrated stroke patients with lower MAP are at high risk. This high-risk subgroup may benefit from rehydration strategies.

## Conclusions

Lower MAP contributes to more severe stroke in patients who are volume contracted but not in those who are euvolemic. The topic of patient hydration status may deserve more attention in the early treatment period after stroke. These results suggest a potentially modifiable risk factor to improve functional outcomes with low cost, broadly available interventions like rehydration.

## Data Availability Statement

Raw data will be made available for any reasonable request of the authors.

## Ethics Statement

The studies involving human participants were reviewed and approved by Institutional Review Board, Johns Hopkins School of Medicine. Written informed consent for participation was not required for this study in accordance with the national legislation and the institutional requirements.

## Author Contributions

All authors listed have made a substantial, direct, and intellectual contribution to the work and approved it for publication.

## Funding

This work was supported by NIH (NINDS) Grants R01NS047691 to AH, R25 NS065729 to AH with supplement to MB; Richard Starr Ross Clinician Scientist Association Grant to MB, K24 AG052573 to RG, and the American Heart Association Career Development Grant to MB.

## Conflict of Interest

The authors declare that the research was conducted in the absence of any commercial or financial relationships that could be construed as a potential conflict of interest.

## Publisher's Note

All claims expressed in this article are solely those of the authors and do not necessarily represent those of their affiliated organizations, or those of the publisher, the editors and the reviewers. Any product that may be evaluated in this article, or claim that may be made by its manufacturer, is not guaranteed or endorsed by the publisher.
